# Leptin and risk factors for atherosclerosis: A review

**DOI:** 10.1097/MD.0000000000036076

**Published:** 2023-11-17

**Authors:** Cheng Wang, Liping Chang, Jia Wang, Libo Xia, Liyuan Cao, Wei Wang, Jianwen Xu, Huize Gao

**Affiliations:** a Affiliated Hospital, Changchun University of Chinese Medicine, Changchun, China; b College of Integrated Traditional Chinese and Western Medicine, Changchun University of Chinese Medicine, Changchun, China; c School of Traditional Chinese Medicine, Changchun University of Chinese Medicine, Changchun, China.

**Keywords:** atherosclerosis, diabetes, inflammation, leptin, obesity, sleep disorder

## Abstract

Leptin is a hormone secreted primarily by adipose tissue. It regulates an organism’s metabolism, energy balance, and body weight through a negative feedback mechanism. When a person or animal has low body fat and little energy, the leptin level in the body decreases, and conversely, when there is an excess of nutrients, the leptin level increases, giving a feeling of satiety. However, when leptin levels are abnormal (too high or too low) for a number of reasons, it can negatively affect your health, inducing inflammatory responses, obesity, and other problems. Many studies have shown that abnormal leptin levels, such as hyperleptinemia, are closely associated with common risk factors for atherosclerosis (AS). This review systematically states the relationship between leptin and common risk factors for AS (inflammation, obesity, diabetes mellitus, hypertension, and sleep disorders) and provides some new thoughts on the future direction of research on both. Because the abnormal level of leptin will have adverse effects on multiple atherosclerotic risk factors, how to regulate the leptin level of patients with AS, and whether we can treat and prevent AS by intervening the leptin level, these may be our new research directions in the future.

## 1. Introduction

Leptin is a hormone discovered in 1994. It is mainly secreted by adipocytes.^[1]^ Leptin can bind to leptin receptor and cross the blood–brain barrier to act on the hypothalamus. It can inhibit appetite and control food intake.^[2]^ In recent years, with the increase of leptin research, it has been found that abnormal leptin levels in the human body also have an important impact on diabetes, obesity, inflammation and other aspects.^[3,4]^ However, obesity, hypertension, inflammatory reaction and other risk factors are common causes of atherosclerosis (AS).^[5,6]^ AS is thought to be the result of a variety of factors characterized by thickening and stiffening of the arterial wall, narrowing of the vessel lumen, and localized fibrous tissue proliferation, lipid accumulation, and calcium deposits.AS is an important cause of coronary heart disease, cerebral infarction and other diseases, which seriously affects the health of patients. At the same time, it also brings huge economic burden to patients. The exact pathogenesis of AS is not well understood, but the risk factors that can lead to its emergence and progression have been investigated for many years. Leptin, as a pleiotropic cytokine, may be closely related to several common risk factors of AS. Therefore, it is of great significance to explore the relationship between leptin abnormalities and as risk factors. This review collates and summarizes the latest research results and related literature of leptin on risk factors of AS, to facilitate the exploration of the relationship between leptin and AS.

## 2. Effect of leptin on risk factors for AS

### 2.1. Leptin and inflammation

Inflammatory changes of endothelial cells play an important role in the process of AS.^[7]^ Leptin can aggregate monocytes, promote the emergence of foam cells, and accelerate the production of proinflammatory cytokines.^[8]^ Elevated leptin levels in patients can directly or indirectly cause dysfunctions of the vascular endothelium, such as the production of large amounts of inflammatory factors and the occurrence of cellular infiltration. In particular, macrophages accumulate under the vascular endothelium and phagocytose oxidatively modified low-density lipoproteins (LDL) to form foam cells, which lead to inflammatory responses through complex cytokines and other pathways. Chronic inflammation of the vascular endothelium can be induced by a variety of mechanisms.^[9]^ The long form (OBRb) and short form (OBRa) of leptin receptor can be expressed by monocytes and macrophages, and induce 2 common inflammatory cytokines: interleukin-6 (IL-6) and tumor necrosis factor α (TNF-α).^[10,11]^ An imbalance between proinflammatory cytokines, such as TNF-a and IL-6, and anti-inflammatory factors leads to the development of chronic inflammation in the vasculature.^[12]^ In addition, eosinophils also secrete inflammatory cytokines under the influence of leptin.^[13]^ T cells also play an important role in the inflammatory response. Studies have shown that leptin affects the functional differentiation of T cells, promoting the production of Th1 and Th17 cytokines, and inhibiting anti-inflammatory Th2 and Treg cells.^[14–17]^ Janus kinase–signal transducer and activator of transcription signaling pathway has a very important effect on the development of AS.^[18]^ Toll like receptor 4 (TLR4) is another important signal that can affect the inflammatory activation and lipid metabolism of AS.^[19]^ Inflammation substances activate the corresponding receptors, regulate the release of inflammatory factors by activating NF-kB through the TLR4 pathway, and stimulate the expression of adhesion factors by vascular endothelial cells.^[20]^ In vitro studies on B cells have shown that leptin stimulates B cells to secrete inflammatory factors (e.g., TNF-α and IL-1) through the Janus kinase and signal transducer and activator of transcription pathways, and that B cells stimulated by leptin have increased expression of inflammatory factors (TNF, TLR4, and IL-6).^[21,22]^ All of the above inflammatory factors are strongly associated with AS.TNF-α and IL-1 can stimulate the liver to secrete C-reactive protein (CRP). CRP is a marker for the development of atherosclerotic inflammation, inhibits endothelial cell repair, and also binds to LDL to promote atherosclerotic plaque formation.^[23,24]^ Leptin can also induce mRNA transcription and stimulate the endothelial cells of coronary arteries to produce CRP.^[25]^ As another inflammatory marker of AS, IL-6 can destroy the endothelial function of blood vessels, increase the adhesion factor of blood vessel walls, and promote the production of Foam cell.^[26]^ In patients with coronary AS, the level of IL-6 is closely related to plaque rupture.^[27]^ In a Cohort study, leptin levels are correlated with IL-6, which may play an important role in the initiation of inflammation.^[28]^ It can be seen that leptin is closely related to the inflammatory response of AS. Several studies have shown that leptin has an inflammatory effect and affects the whole process of AS.

### 2.2. Leptin and obesity

As is well known, obesity is one of the important risk factors for AS. Obesity can induce vascular inflammation, affect vascular endothelial function, and aggravate the degree of endothelial damage.^[29–31]^ After vascular endothelial damage caused by obesity or overweight, LDL enters the damaged vessels, attracts monocytes to aggregate, and forms foam cells to induce smooth muscle overproliferation and secretion.^[32]^ A study showed that obesity can make AS progress even in patients with hyperlipidemia treated with statins.^[33]^ Leptin, as a hormone secreted by adipocytes, is closely related to obesity. Early studies have shown that neuropeptide Y in the arcuate nucleus of the hypothalamus (ARC) can be inhibited by leptin, regulating body weight and energy metabolism.^[34]^ When ARC and other thalamic nuclei with OBRb are damaged, diseased rats may experience overeating and obesity.^[35]^ In a cross-sectional study related to leptin and obesity, it was found that serum leptin levels in obese and overweight patients were significantly higher than those in healthy subjects.^[36]^ Since leptin can suppress appetite and regulate energy metabolism, why is high leptin levels closely related to obesity? This is because many obese people will appear leptin resistance, that is, the sensitivity to leptin is reduced or unresponsive. Therefore, hyperleptinemia has also become a feature of most obese people. The mechanism of hyperleptinemia and leptin resistance is complex. In addition to the congenital leptin deficiency defect, a major reason is the decreased expression of leptin receptor or the disturbance of signal transduction caused by various reasons.^[37,38]^ For example, obesity can lead to endoplasmic reticulum stress and induce chronic inflammatory state, thus leading to the attenuation of OBRb signaling pathway.^[39]^ Stimulation of external factors, such as Circadian rhythm and diet structure, may also affect the synthesis and expression of leptin, leading to leptin resistance and obesity.^[40]^ It is worth noting that hyperleptinemia does not only contribute to AS through obesity; a recent study has shown that hyperleptinemia can exacerbate AS directly by depressing vascular tone and increasing atherosclerotic plaque.^[41]^

In the obese and overweight population, lipid abnormalities should receive special attention. Dyslipidemia not only contributes to AS, but is also strongly associated with leptin levels. As a manifestation of abnormal lipid metabolism, impaired cholesterol homeostasis can be one of the factors that trigger and exacerbate AS.^[42]^ Hyperleptinemia inhibits the transcription factor SREBP2 (Sterol Regulatory Element Binding Protein 2), which leads to a decrease in the expression of the LDLR (Low Density Lipoprotein Receptor), causing hypercholesterolemia.^[43]^ The relationship between leptin and cholesterol was also demonstrated in an animal study where rats had significantly higher total cholesterol levels after being injected subcutaneously with leptin for 8 days.^[44]^ Triglycerides (TG) are another important component of blood lipids, and increased TG levels have been an independent risk factor for AS.^[45]^ Leptin stimulates hepatic secretion of TG via the brain-vagus-hepatic axis.^[46]^ In summary, abnormal leptin levels are closely associated with obesity and dyslipidemia. Leptin resistance may be the key to this set of problems.

### 2.3. Leptin and diabetes

**Figure 1.**
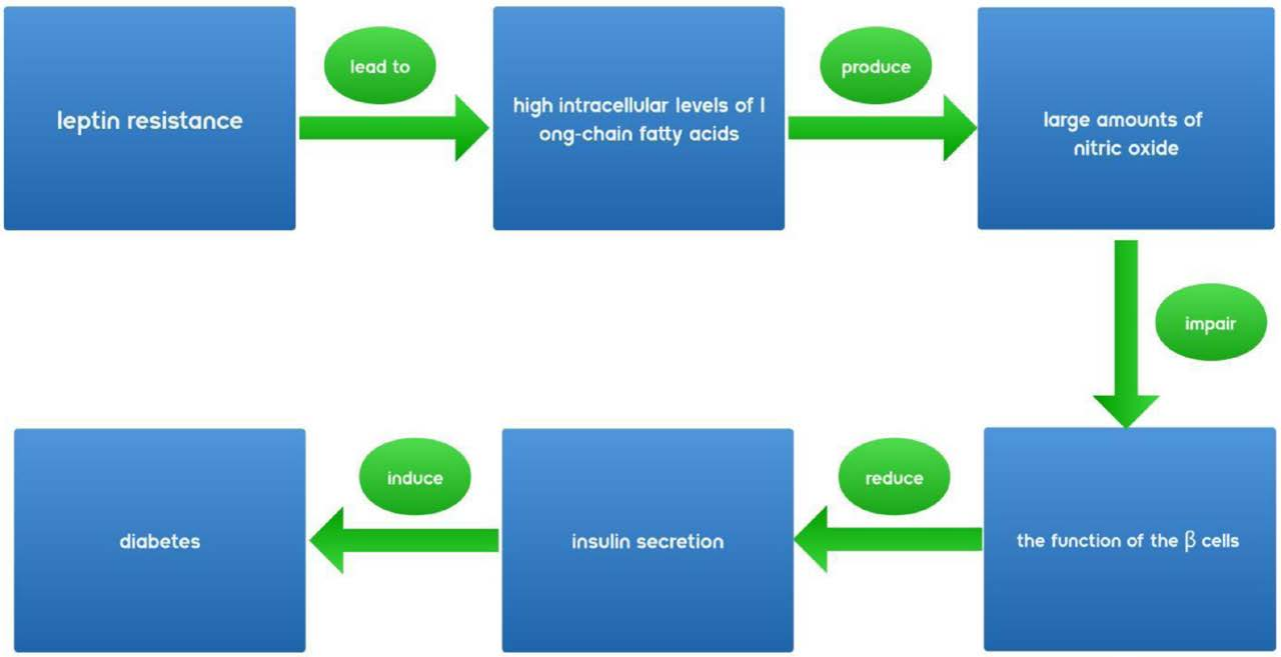
Schematic diagram of leptin resistance inducing or exacerbating diabetes.

The relationship between diabetes and AS has been explored for many years. As an independent risk factor for AS, diabetes mellitus is thought to affect AS through a variety of pathways including effects on lipid metabolism, increased oxidative stress, activation of protein kinase C, and chronic inflammation.^[47]^ In a control study based on community population, researchers measured the cardiometabolic spectrum of 3908 subjects and scanned bilateral carotid arteries. Through statistical analysis of the prevalence, number of carotid plaques, carotid plaque score and other items, it was concluded that the prevalence of carotid plaques in diabetic patients was higher.^[48]^ A prospective study in an elderly population suggests that circulating leptin levels in humans are strongly associated with the development of diabetes and can be used to predict diabetes risk.^[49]^ A recent clinical study also suggests that higher-than-normal leptin levels in patients with type 2 diabetes may have an impact on short-term glycemic control, as well as a strong correlation between patients’ hyperleptinemia and the incidence of diabetic complications.^[50]^ Leptin affects glucose metabolism through the central nervous system and regulates its homeostasis. Although it has been suggested that moderate amounts of leptin can lower blood glucose and increase the body’s sensitivity to insulin, the effects of hyperleptinemia and leptin deficiency, on glucose metabolism and diabetes are mostly negative.^[51,52]^ Insulin resistance (IR) or abnormal insulin secretion are the main causes of diabetes, and coincidentally, it has been shown that leptin appears to have an effect on both. In a case–control study, it was found that diabetic patients in the experimental group showed a significant correlation between serum leptin levels and insulin levels compared to controls in the healthy population (n = 73, *P* < .001), and it was concluded that serum leptin levels could be an important predictor of IR.^[53]^ Hyperleptinemia can lead to exacerbation of IR and impaired glucose tolerance^[54,55]^ Animal experiments suggest that hyperleptinemia’s effect on IR may be due to the fact that both leptin and insulin share a common ARC neuronal response, and that when leptin receptor signaling is continuously activated in the ARC, it increases the expression of the insulin receptor phosphatase, PTP1B, which blocks insulin signaling and reduces the inhibitory effect of insulin on glucose production.^[56,57]^ It has also been shown that leptin resistance leads to excessively high intracellular levels of long-chain fatty acids, which leads to the production of large amounts of nitric oxide, which impairs the function of the β cells and reduces insulin secretion, inducing diabetes or making it worse^[58]^ (as shown in Fig. 1). Thus, abnormalities in leptin are closely related to diabetes, and high leptin levels also have the potential to adversely affect cardiovascular autonomic function in diabetic patients, which may be another way in which leptin affects AS through diabetes.^[59]^

### 2.4. Leptin and hypertension

The relationship between AS and hypertension has always been closely monitored by researchers, and it has been proven in both clinical and basic studies that hypertension can have adverse effects on AS.^[60]^ A study by Gonzalez-Guerrad et al showed that even a small increase in blood pressure alone can have some effect on the appearance of AS plaques.^[61]^

For the relationship between leptin and blood pressure, in an earlier observational study, leptin was found to be positively correlated with mean blood pressure in subjects with hypertension (n = 67, *P* < .05).^[62]^ The close relationship between leptin and blood pressure can also be demonstrated in rodents. In obese rats, it can be observed when an elevation in leptin levels causes an increase in blood pressure. However, when leptin is deficient or the leptin receptor is absent, this effect will not be present.^[63]^ The effect of leptin on increased blood pressure may be mediated by the sympathetic nervous system. Downstream effects mediated by leptin signaling in the hypothalamus result in activation of nigroportin receptors on spinal sympathetic neurons. This activation of the sympathetic nervous system then causes the renin angiotensin system to respond, ultimately leading to increased blood pressure.^[64]^ A cross-sectional study also suggests that the increase in arterial blood pressure is caused by leptin’s activation of hypothalamic sympathetic nerves.^[65]^ Gruber et al suggested that hyperleptinemia dilate microvascular structures in the brain that regulate hemodynamic homeostasis, hyperleptinemia also affects the HIF1α-VEGF (hypoxia inducible factor 1α-vascular endothelial growth factor) signal transduction of hypothalamic astrocytes, and its cascade reaction will promote the occurrence of hypertension.^[66]^ In another study, leptin-overexpressing transgenic rats, independent of adipose tissue, still developed sympathetically mediated hypertension even though the rats were lean.^[67]^ This suggests that high levels of leptin may cause hypertension through the sympathetic system alone, rather than having an effect by inducing obesity. Leptin receptor can be expressed in adrenal glomerular cells, and hyperaldosteronemia and hyperleptinemia often occur simultaneously in obese patients. Some studies believe that high leptin levels will lead to increased aldosterone secretion, and then promote the occurrence and development of hypertension.^[67–69]^ In summary, pathologically increased leptin levels can make blood pressure higher, and this effect can be due to a number of reasons. In a case–control study (n = 153), researchers divided the subjects into 4 groups: normal health group, newly diagnosed untreated simple hypertension group, newly diagnosed untreated obese hypertension group, and newly diagnosed obesity group. The leptin level, body mass index, blood pressure, waist circumference, and other information of each group were measured and recorded, and the multiple linear regression analysis was carried out. It was concluded that the effect of leptin on hypertension may not be direct, but play an additive effect through a variety of regulatory mechanisms.^[70]^

### 2.5. Leptin and sleep disorders

Sleep disorders include obstructive sleep apnea (OSA), insomnia, inadequate sleep duration, abnormal awakenings, and more. Sleep disorders have been shown in several studies to increase the risk of AS, for example, insufficient sleep duration leads to thickening of the middle carotid artery intima-media, and also exacerbates coronary artery calcification.^[71,72]^ OSA, in particular, has emerged as an independent risk factor for AS.^[73]^ Leptin influences and regulates the body’s circadian rhythms and its secretion has a certain diurnal pattern.^[74,75]^ Studies have pointed out that the suprachiasmatic nucleus of the hypothalamus can respond to light stimulation, and it should be the key to regulate the circadian rhythm of leptin.Leptin deficient mice (ob/ob) have reduced sensitivity to light and circadian rhythms are affected.^[76,77]^ Many observational studies have proved that leptin is closely related to sleep disorders. In a randomized controlled experiment, the experimental group was OSA patients (n = 21), and the control group was non OSA patients. The leptin level of OSA patients was significantly higher than that of the control group.^[78]^ In the study of Hirota et al, the sleep structure of diabetic patients was evaluated using electroencephalogram, and the fasting serum leptin level of patients was measured (n = 113). It was concluded that leptin level was positively related to sleep quality.^[79]^ However, some of the conclusions exploring the relationship between leptin and sleep are contradictory, with Hayes et al showing that elevated leptin is strongly associated with decreased sleep, in contrast to another view that hypoleptinemia is an important cause of decreased sleep.^[80,81]^ OSA is the risk factor most strongly associated with AS in sleep disorders and can lead to intermittent hypoxia, increased blood viscosity and monocyte adhesion.^[82,83]^ In addition to circadian rhythms, leptin may have an effect on OSA through musculo-neural responses in the upper airway.^[84]^ In a meta-analysis related to OSA, the results showed that the leptin level of OSA patients in the experimental group was increased (n = 469, *P* < .001).^[85]^ Thus, abnormal levels of leptin do adversely affect sleep disorders. Whether this adverse effect is mediated by obesity or whether leptin acts independently is controversial and still needs to be explored in more studies.^[84,86]^

## 3. Summary and outlook

**Figure 2.**
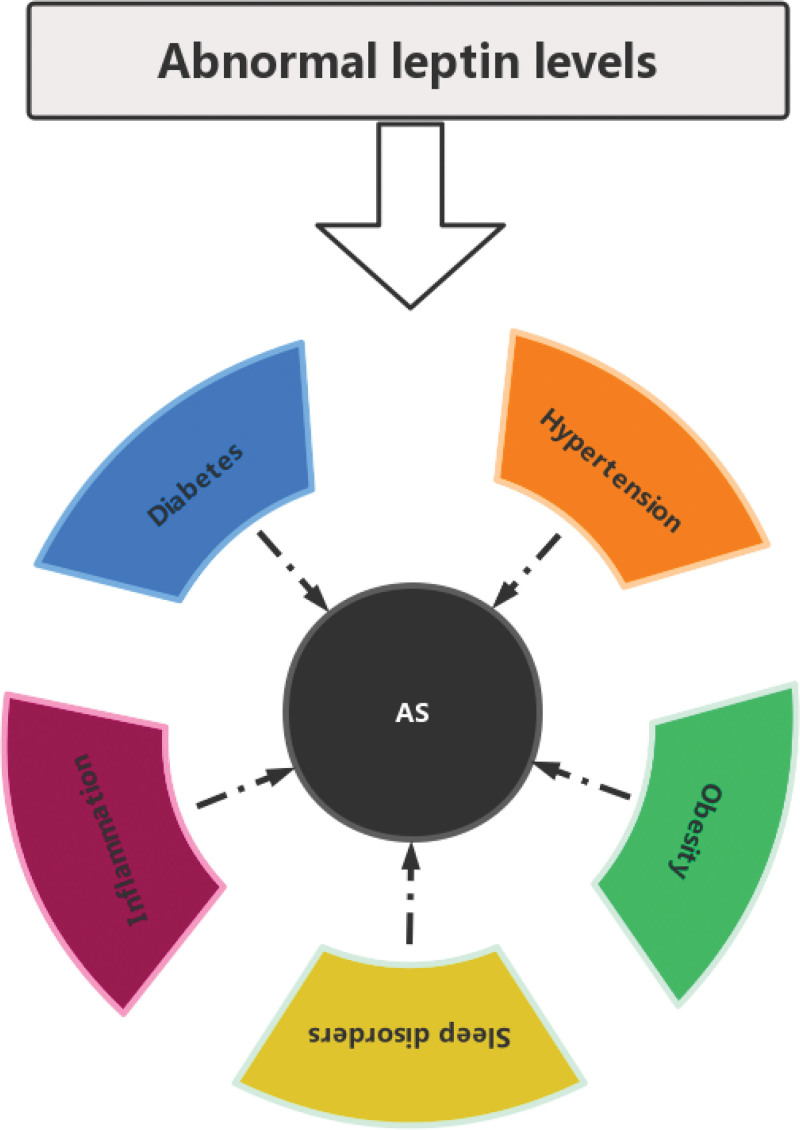
Map of the relationship between abnormal leptin levels and common risk factors for AS. AS = atherosclerosis.

In summary, abnormal leptin levels can have an impact on inflammation, obesity, diabetes, and other common risk factors for AS, as shown in Figure 2. However, there are still several issues that deserve our continued reflection and exploration.

1. It has been suggested that leptin can directly affect endothelial proliferation, vascular smooth muscle proliferation and migration, and macrophage proliferation, and induce the development and exacerbation of AS through the interaction of leptin and its receptors.^[87,88]^ So, is the adverse effect of leptin on AS a direct effect on the arterial vasculature, or is it through a combination of risk factors that exacerbate AS?
2. There are multiple risk factors for AS, and in addition to several common risk factors listed in this review, some studies suggest that oxidative stress, mood disorders (e.g., anxiety, depression), and cognitive disorders (e.g., Alzheimer disease, dementia) may also have an adverse effect on AS.^[89–91]^ Abnormal leptin levels that are too high or too low have also been associated with oxidative stress, cognitive impairment, and mood disorders. However, it has been shown that these adverse effects also appear to be mediated by obesity, diabetes, or inflammation, and more research is needed to support whether leptin directly contributes to these risk factors and ultimately affects AS.^[92–94]^
3. The adverse effects of leptin are all brought about by its levels being too high or too low. For example, leptin treatment can also play a positive protective effect on the blood vessels of malnourished rats.^[95]^ Therefore, we cannot define leptin as a “bad” substance. How to maintain leptin levels at a steady state in the body is a question that is well worth investigating.
4. Leptin is closely related to the risk factors of AS and to AS itself, then we should pay attention to the impact that leptin has in this process when it comes to the prevention and treatment of AS. Can we capitalize on the positive effects of leptin on AS and circumvent the negative effects of leptin in the future treatment of AS?
5. Clinical studies on the treatment of abnormal leptin levels suggest that exercise may be one way to lower leptin levels. In a randomized controlled trial it was shown that adolescents at risk for diabetes in the intervention group (n = 22) had reduced leptin levels after 3 months of participation in a physical activity program.^[96]^ In addition, acupuncture also down-regulated leptin levels in obese rats in animal studies.^[97]^ So in the treatment of high leptin levels, in addition to exercise and acupuncture, can we develop a new drug to help better down-regulate abnormal leptin levels?
6. Heart failure deserves close attention as one of the serious complications of AS. It has been shown that high expression of leptin levels impairs ventricular diastolic function in patients with coronary artery disease and also exacerbates the condition of patients with heart failure, and that this adverse effect is not associated with an immune response.^[98,99]^ So could leptin therapy be a new approach to treating heart failure and other complications of AS?

The above problems still need a lot of basic research and clinical research to continue to explore.

## Author contributions

**Conceptualization:** Cheng Wang, Liping Chang, Libo Xia.

**Data curation:** Cheng Wang, Jia Wang, Libo Xia.

**Formal analysis:** Cheng Wang, Libo Xia.

**Funding acquisition:** Liping Chang.

**Investigation:** Cheng Wang, Jia Wang, Liyuan Cao, Wei Wang, Huize Gao.

**Methodology:** Cheng Wang, Liping Chang.

**Project administration:** Libo Xia.

**Resources:** Jia Wang, Liyuan Cao, Jianwen Xu.

**Software:** Liyuan Cao, Wei Wang.

**Supervision:** Liping Chang.

**Validation:** Wei Wang, Jianwen Xu, Huize Gao.

**Visualization:** Jia Wang.

**Writing – original draft:** Cheng Wang.

**Writing – review & editing:** Liping Chang.
